# Aberrant Brain Network Efficiency in Parkinson’s Disease Patients with Tremor: A Multi-Modality Study

**DOI:** 10.3389/fnagi.2015.00169

**Published:** 2015-08-31

**Authors:** Delong Zhang, Jinhui Wang, Xian Liu, Jun Chen, Bo Liu

**Affiliations:** ^1^Department of Radiology, Guangdong Provincial Hospital of Chinese Medicine, Guangzhou, China; ^2^Guangzhou University of Chinese Medicine Postdoctoral Mobile Research Station, Guangzhou, China; ^3^Center for Cognition and Brain Disorders, Hangzhou Normal University, Hangzhou, China; ^4^Zhejiang Key Laboratory for Research in Assessment of Cognitive Impairments, Hangzhou, China

**Keywords:** Parkinson’s disease, resting-state network, individual morphological network, multivariate analysis, tremor

## Abstract

The coordination of spontaneous brain activity is widely enhanced relative to compensation activity in Parkinson’s disease (PD) with tremor; however, the associated topological organization remains unclear. This study collected magnetic resonance imaging data from 36 participants [i.e., 16 PD patients and 20 matched normal controls (NCs)] and constructed wavelet-based functional and morphological brain networks for individual participants. Graph-based network analysis indicated that the information translation efficiency in the functional brain network was disrupted within the wavelet scale 2 (i.e., 0.063–0.125 Hz) in PD patients. Compared with the NCs, the network local efficiency was decreased and the network global efficiency was increased in PD patients. Network local efficiency could effectively discriminate PD patients from the NCs using multivariate pattern analysis, and could also describe the variability of tremor based on a multiple linear regression model (MLRM). However, these observations were not identified in the network global efficiency. Notably, the global and local efficiency were both significantly increased in the morphological brain network of PD patients. We further found that the global and local network efficiency both worked well on PD classifications (i.e., using MVPA) and clinical performance descriptions (i.e., using MLRM). More importantly, functional and morphological brain networks were highly associated in terms of network local efficiency in PD patients. This study sheds lights on network disorganization in PD with tremor and helps for understanding the neural basis underlying this type of PD.

## Introduction

The spontaneous activity of human brain is highly structured in which anatomical regions interact within a network (Schnitzler and Gross, 2005; Bullmore and Sporns, 2012). The topological organization of brain networks can be depicted and characterized by the concept of graph theory (Bullmore and Sporns, 2009; Park and Friston, 2013). Graph-based network analysis demonstrates an optimized topology in brain networks, with efficient information transmission and exchange (Bassett and Bullmore, 2006). This normal network organization is disrupted in certain brain diseases (Bassett and Bullmore, 2009; He et al., 2009a; Zhang et al., 2011; Wang et al., 2013). The disrupted network topology has been linked to the neural basis underlying brain dysfunction, which has provided a new avenue to elucidate the essence of brain disorders.

Parkinson’s disease (PD) is a chronic neurodegenerative disorder with an array of motor symptoms, such as resting tremor, akinesia, and rigidity (Deuschl et al., 2000; Lees et al., 2009). Neuroimaging studies have shown that resting-state functional connectivity (RSFC) (Hacker et al., 2012; Tessitore et al., 2012) and spatial topology (Skidmore et al., 2011) are disrupted in the patients with PD. In particular, resting-state network organization exhibits lower network efficiency in PD patients relative to normal controls (NCs) (Skidmore et al., 2011; Gottlich et al., 2013), and the reduction of the network global efficiency longitudinally accumulates with PD progression (Olde Dubbelink et al., 2014). By contrast, many studies have found that PD patients with tremor exhibit widespread increased RSFC compared with NCs (Helmich et al., 2011, 2012; Prodoehl et al., 2013; Zhang et al., 2015). This difference may stem from the fact that the pathophysiology of PD is heterogeneities across clinical symptoms (Thenganatt and Jankovic, 2014). Despite the degeneration of dopaminergic neurons in the basal ganglia being linked to reduced brain network efficiency, there are salient compensation events in PD patients with tremor (Helmich et al., 2012; Zhang et al., 2015). These compensation events may lead PD patients with tremors to be a unique PD state with widespread increases in functional connectivity (Helmich et al., 2012; Zhang et al., 2015). Moreover, the compensation mechanisms also benefit the PD patients with tremor, who show fewer cortical lesions (Helmich et al., 2012) and slower development of dementia-like cognitive dysfunction (Aarsland et al., 2003; Williams-Gray et al., 2007; Helmich et al., 2012). Given the reduced network efficiency documented in PD patients, it is necessary to further explore the brain network topology underlying the compensation in PD patients with tremor.

The present study examined the brain network topology of PD patients with tremor using functional and individual morphological brain networks. We set out to assess (1) functional brain network topologies based on wavelet-based RSFC in PD patients with tremor relative to NCs, (2) the spatial organization of individual PD morphological brain networks, and (3) the linkage between the two types of brain networks and the patient’s clinical performance. We collected resting-state functional magnetic resonance imaging (rs-fMRI) data and anatomical T1 data from 16 PD patients with tremor and 20 matched NCs. Whole-brain functional brain networks were constructed using wavelet-based RSFC, and cortical morphological networks were constructed using the inter-regional gray matter (GM) similar analysis approach. The topological organization of the functional and morphological brain networks and their relationships were investigated in terms of network efficiency in PD patients relative to the NCs.

## Materials and Methods

### Participants

Sixteen right-handed PD patients with resting tremor (nine males and seven females) were recruited from the Second Affiliated Hospital of Guangzhou University of Traditional Chinese Medicine. A detailed clinical assessment, including history, physical, neurological, and neuropsychological examinations, including the Unified Parkinson’s Disease Rating Scale (UPDRS I–IV), Mini-Mental State Examination (MMSE), and Hoehn and Yahr Scale (H–Y stage), were performed on each patient. The patients were diagnosed according to UK PD Brain Bank Criteria (Gibb and Lees, 1988). All the PD patients had resting tremor with (Skidmore et al., 2011) or without (Bullmore and Sporns, 2012) action or postural tremor. These patients exhibited bilateral hand tremor (i.e., eight patients with right hand tremor and two with left hand tremor) or bilateral hand tremor (i.e., six patients). The resting tremor level was evaluated by using the sub-scale item 20 of UPDRS III (Krack et al., 1997, 1998; Mahlknecht et al., 2015). The current patients had different levels of resting tremor (eight tremor-dominant patients and eight non-tremor-dominant patients). The exclusion criteria included a history of other neurological or psychiatric conditions, including secondary Parkinsonism, atypical parkinsonian disease, advanced PD stages (H–Y, 4–5), dementia (MMSE score <24). There were no any substance dependence and head trauma in all these patients. In addition, a total of 20 age-, gender-, and education-matched NCs (11 males and 9 females) were collected for the present study. This study was approved by the Institutional Review Board of the Guangzhou University of Traditional Chinese Medicine. All the participants gave written informed consent for the study. The detailed clinical and demographic information for all participants is shown in Table 1.

**Table 1:** **Demographics and clinical characteristics of the participants**.

	NC (*n* **=** 20)	PD (*n* **=** 16)	*p* Value
Age (years)	42–78 (59.2 ± 8.7)	37–81 (60.5 ± 11.8)	0.37^b^
Gender (M/F)	11/9	9/7	0.90^a^
Illness duration (years)	–	0.42–6 (2.5 ± 1.7)	–
MMSE	–	29.0–30 (29.8 ± 0.05)	–
UPDRS	–	4–49 (27.3 ± 14.3)	–
H–Y	–	1–3 (2.25 ± 0.91)	–
Tremor level	–	1–4 (2 ± 0.85)	–

*Data are presented as minimum–maximum (Mean ± SD)*.

*PD, Parkinson’s disease; NC, nealthy control; MMSE, Mini-Mental State Examination; UPDRS, Unified Parkinson’s Disease Rating Scale; H–Y, Hoehn and Yahr Scale; tremor level, resting tremor score of item 20 in UPDRS III*.

*The value was obtained using a two-tail Pearson chi-square test*.

*The value was obtained using two-sample two-tail tests*.

### Image acquisition

All participants were scanned using a 1.5-T Siemens scanner at the department of radiology of the Second Affiliated Hospital of Guangzhou University of Traditional Chinese Medicine. Rs-fMRI data were collected using an echo-planar imaging sequence: 30 axial slices; repetition time (TR) = 2000 ms; echo time (TE) = 39 ms; slice thickness = 4 mm; gap = 1 mm; flip angle (FA) = 90°; matrix = 64 × 64; field of view (FOV) = 240 mm × 240 mm. Participants lay quietly in the scanner with their eyes closed and foam padding was used to restrict head motion as far as possible during data acquisition. In total, 180 volumes were obtained for each participant. We acquired 3D structural images using a T1-weighted MP-RAGE sequence: 192 sagittal slices; TR = 1160 ms; TE = 4.21 ms; inversion time = 600 ms; slice thickness = 0.9 mm; no gap; FA = 15°; matrix = 512 × 512; FOV = 256 mm × 256 mm.

### Data preprocessing

Resting-state functional magnetic resonance imaging data preprocessing was performed with the GRETNA toolbox (Wang et al., 2015) based on SPM8^1^. Briefly, functional preprocessing included the following: (1) the first five volumes were discarded to allow for scanner stabilization; (2) the time offsets between slices as well as geometrical displacements due to head movement of the functional images were corrected. According to the criterion of a displacement >3 mm or an angular rotation >3° in any direction, none of the participants were excluded. The summary scalars of both gross (maximum and root mean square) and micro (mean frame-wise displacement) head motion were matched between the two groups (all *p* > 0.15); (3) Using an optimum 12-parameter affine transformation and non-linear deformations, all corrected functional data were then normalized to the Montreal Neurological Institute (MNI) space, and then resampled to 3-mm isotropic resolution; (4) the effects of low-frequency drift and high-frequency physiological noise of the resulting images were further reduced in term of the temporally band-pass filtered (0.01–0.1 Hz); and (5) after removing the linear trend, several nuisance signals, including 24-parameter head-motion profiles, mean white matter (WM), and cerebrospinal fluid (CSF) time series, were also regressed out from each voxel’s time course.

Structural data (3D T1-weighted anatomical images) preprocessing was implemented by the VBM8 toolbox in SPM8^2^. The structural processing steps included the following: (1) we firstly applied an adaptive Maximum A Posterior (MAP) technique to segment the structural images into GM, WM, and CSF; (2) then, the GM maps were normalized (using a DARTEL approach) into the MNI space; (3) in addition, the non-linear modulation of GM maps were used to compensate for spatial normalization effects; and (4) the GM maps were spatially smoothed using a 6-mm full width at half maximum Gaussian kernel.

### Network construction

#### Wavelet-Based Functional Network

A wavelet-based functional network (Wang et al., 2013) was constructed for each individual participant. For functional network construction, the nodes were defined using a previous brain atlas with 264 putative functional areas, defined using neurobiological principles (Power et al., 2011). The nodes were distributed across the cerebral cortex, subcortical structures, and the cerebellum. For the edges, we applied the maximal overlap discrete wavelet transform method (Percival and Walden, 2000) to obtain the wavelet coefficients; then, the interregional RSFC was calculated as the edges by calculating the Pearson correlation between any pair of ROIs in wavelet coefficients. In this study, the brain functional networks related to four wavelet scales (scale 1, 0.125–0.250 Hz; scale 2, 0.063–0.125 Hz; scale 3, 0.031–0.063 Hz; and scale 4, 0.016–0.031 Hz) were constructed and investigated.

#### Individual Morphological Network

To construct the morphological network, the node definition was the same as the functional network. The edges of the morphological network represented the GM similarity between nodes. In this study, the intracortical similarity was measured by using a seed cube similarity approach (Tijms et al., 2012). Using this method, the correlation coefficient of the regions on aspect of three dimensional structure of the cortex intact, geometrical information, and the GM values in the voxels of defined cubes were measured. It should be noted that the maximum correlation value was computed over different rotations of the seed cube, in which the regions with zero variance in GM values were excluded (average across all subjects, 0.01%). Here, only positive similarity values survived this threshold. Finally, the similarity values were binarized after determining a threshold for each individual graph.

### Network analysis

The network analysis was the same for the two types of brain networks and was calculated after the network construction procedure.

#### Thresholding Procedure

A sparsity threshold was applied to measure the individual correlation matrices. Considering it is different to select a single threshold, an empirically thresholded scope was used on the wide range of 0.02 ≤ sparsity ≤ 0.4 (interval = 0.02). We calculated global and local efficiency of the resultant networks at each sparsity (Latora and Marchiori, 2003; Achard and Bullmore, 2007). Similar to previous studies (He et al., 2009b; Zhang et al., 2011), the area under the curve (AUC) for each network metric (global and local efficiency) was calculated to obtain a summarized scalar.

#### Network Metrics

Network efficiency has been widely used to depict parallel information flow within brain network (Latora and Marchiori, 2001; Achard and Bullmore, 2007). For the constructed brain morphological networks, we calculated the network efficiency to characterize the brain topological organization. Here, the network efficiency was described in the context of a binary network *G* with *N* nodes and *K* edges. The global efficiency for a network *G* is defined as:

(1) $$E_{\text{glob}}(G)=\frac{1}{N(N-1)}\sum_{\text{i}\neq \text{j}\in \text{G}}\frac{1}{d_{\text{ij}}}$$

where *d*_ij_ is the shortest path length between node i and node j in *G* and is calculated as the smallest sum of edge lengths throughout all possible paths from node i and node j. Global efficiency measures the parallel information transmission ability over the whole network. Instead, local efficiency measures the capability of information exchange for each subgraph. And the local efficiency of *G* is measured as:

(2) $$E_{\text{loc}}(G)=\frac{1}{N}\sum_{\text{i}\in \text{G}}E_{\text{glob}}(G_{\text{i}})$$

where *E*_glob_(*G*_i_) is the global efficiency of *G*_i_, the subgraph comprised the neighbors of the node i.

Global and local efficiency were normalized by the related mean metrics of the 100 random networks. These random networks obtained the same number of nodes, edges, and degree distributions as the real brain networks. On aspect of the normalized network efficiency, small-world architecture of the brain network was defined as the normalized local efficiency is larger than 1 and the normalized global efficiency is approximately equal to 1.

The nodal efficiency of a given node i is computed as (Achard and Bullmore, 2007)

(3) $$e_{\text{i}}=\frac{1}{N-1}\sum_{\text{j}\neq \text{i}\in \text{G}}\frac{1}{d_{\text{ij}}}$$

Nodal efficiency reflects the information propagation ability of a node with the others within a network.

The network metrics were calculated using the GRETNA toolbox (Wang et al., 2015). The visualization of brain ­networks was implemented by the BrainNet Viewer (Xia et al., 2013).

### Statistical analysis

Non-parametric permutation tests (10,000 permutations) (Bullmore et al., 1999; He et al., 2008) were used to test differences in between-group brain network metrics. Gender and age were treated as unconcerned covariates for comparisons. For graph-based metrics, a false-positive correction threshold *p* = 1/*N* (*N* = 264) was used for multiple comparison correction (Bassett et al., 2009; Lynall et al., 2010).

A partial correlation approach was used to calculate the association between the network properties and clinical variables. Here, gender and age were used as covariates in the calculation.

### Multivariate pattern analysis

This study applied a multivariate pattern analysis (MVPA) method to explore whether the network local/global efficiency was able to distinguish the PD patients with tremor from the NCs. The brain network nodal efficiency was used as the discriminative feature, and the maximum uncertainty linear discriminate analysis (MLDA) (Dai et al., 2012) was the classifier. Here, the linear classifier was validated by using a leave-one-out cross-validation (LOOCV) approach. A feature selection based on non-parametric permutation tests (*p* < 0.01, uncorrected) was used to reduce the data dimensions. The network nodes with significant between-group differences in network efficiency were selected to form a discriminative pattern. Finally, the labels of samples (PD vs. NC) relative to the discriminative pattern were random disrupted and the classifier validation procedure was repeated 100 times. The distribution of the classifier performance with random labels was used to calculate the *z*-score value, which was used to infer the significance of the classifier performance.

### Multiple linear regressions

A multiple linear regression model (MLRM) using least squares (“regress” in Matlab) was further applied to explore the correlation between brain network efficiency and clinical behavior, as well as the network efficiency between the functional and morphological brain. For regression analysis, clinical performance (e.g., tremor) was used as the response observation and the network nodal efficiency (e.g., local efficiency) was the predictor variable. For the exploration of the correlation between the two types of brain networks, brain functional network local (global) efficiency was used as the response observations, and the morphological network nodal local (global) efficiency was used as the predictor variable, and vice versa. The statistical attribute was used to validate the performance of the regress model. Here, the predictor variables were limited to those with significant correlations (Pearson correlation, *p* < 0.01) with the response observations.

## Results

### Global parameters of brain networks

**Figure 1:**
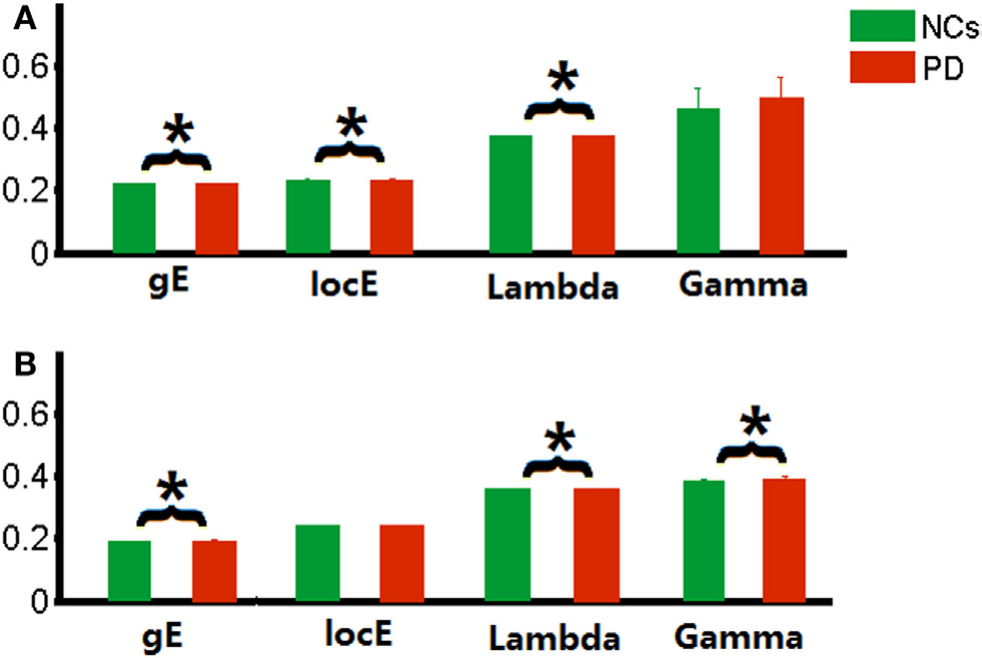
**Global parameters of brain networks**. **(A)** functional brain network related to wavelet scale 2; **(B)** individual morphological brain network. *p < 0.05.

Relative to matched random networks, the network efficiency analysis revealed that a larger local efficiency but approximately equal global efficiency (i.e., small-world organization attribute) was observed in the PD functional brain network. However, statistical comparisons revealed significant differences in the network efficiency between the two groups. The PD patients showed significantly decreased local efficiency (*p* = 0.02), increased global efficiency (*p* = 0.01), and normal global efficiency (*p* = 0.01) in the functional networks (Figure 1A), compared to the NC group.

Similarly, small-world organization was also found in the morphological networks of PD patients. Increased global efficiency (*p* = 0.04), increased normalized global efficiency (*p* = 0.02), and local efficiency (*p* = 0.02) were also observed in the morphological networks of PD patients (Figure 1B).

### Regional parameters of brain networks

Compared with the NCs, altered functional brain network efficiency was observed in the PD patients with tremor. To further localize the brain regions that drive the overall change, we compared nodal efficiency for each node between groups. For local efficiency, decreased nodal local efficiency was observed in the left superior paracentral lobule cortex in PD patients, and increased nodal local efficiency in the left inferior paracentral lobule cortex and the right post cingulated cortex. For global efficiency, the decreased nodal global efficiency and increased nodal global efficiency were found in the right inferior frontal gyrus (triangular part) and the right superior frontal gyrus (orbital part) in PD patients, respectively.

**Figure 2:**
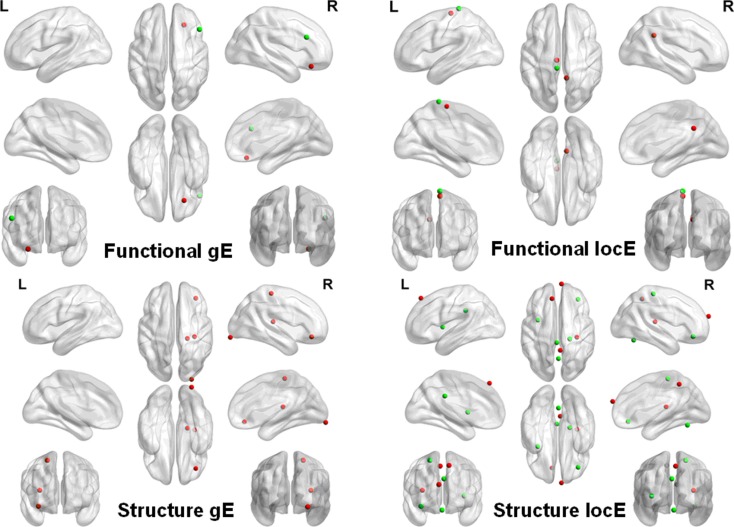
**Network nodal efficiency difference between the PD and NC groups**. L, the left hemisphere; R, the right hemisphere; gE, global efficiency; locE, local efficiency; red color indicates increased efficiency; and green color indicates decreased efficiency in PD patients.

Similarly, we also found that the morphological networks of the PD patients showed significantly increased nodal local efficiency in the right inferior frontal gyrus (orbital part) and precentral gyrus, and left insula and post cingulated cortex, and cerebellum (i.e., Vermis_6). Decreased local nodal efficiency was found in the right Heschl gyrus and precuneus gyrus, and bilateral medial superior frontal gyrus in PD patients. Increased global nodal efficiency was found in regions, including the right inferior occipital cortex, inferior frontal gyrus (orbital part), precental gyrus, and Heschl gyrus. The details are shown in Figure 2.

### Discriminant analysis

The network nodal efficiency was investigated to explore whether it could classify PD patients from NCs. We found that the nodal local efficiency in the functional brain network could effectively discriminate the two groups (Accuracy = 0.81, Sensitivity = 0.88, Specificity = 0.75), which was significantly above the random level (*z* = 2.70). The discriminate regions were located in the left inferior occipital and paracentral lobule, and the right postcentral and post cingulum cortex. However, we did not find sufficient discriminative information in the global nodal efficiency to classify the two groups (Accuracy = 0.63, Sensitivity = 0.68, Specificity = 0.60), which was still at the chance level.

**Figure 3:**
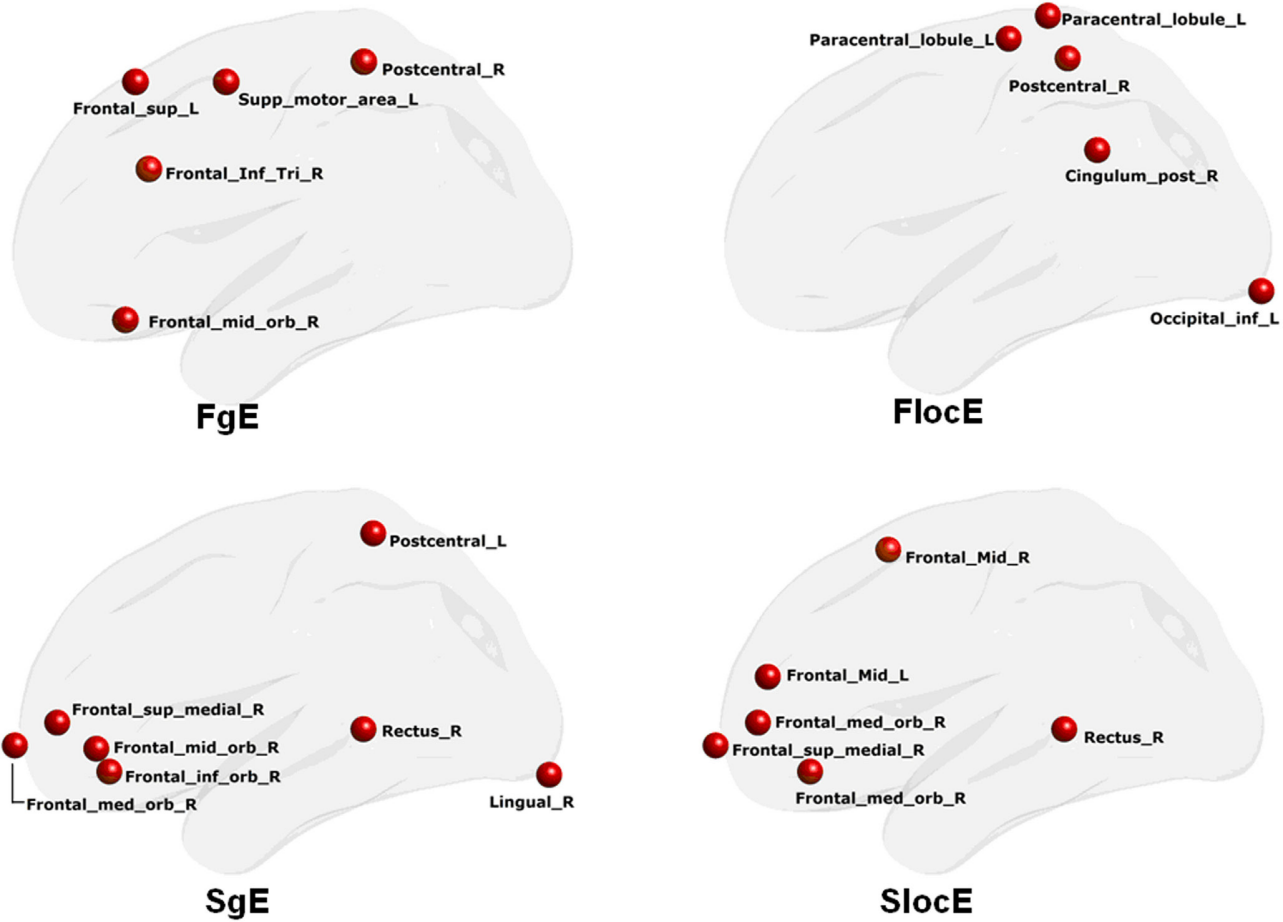
**Discriminative regions that classify PD patients from NCs**. FgE, functional network global efficiency; FlocE, functional network local efficiency; SgE, structure network global efficiency; SlocE, structure network local efficiency.

We also found that the local nodal efficiency in the morphological network could significantly discriminate the two groups (accuracy = 0.77, sensitivity = 0.81, specificity = 0.74) (*z* = 2.53). The regions were located at the right inferior (arbitral part)/medial (arbitral part)/superior/frontal gyrus, and the bilateral middle frontal gyrus. Nodal global efficiency could also discriminate the two groups (accuracy = 0.89, sensitivity = 0.94, and specificity = 0.84) (*z* = 3.51). The discriminative regions were found at the right lingual, rectus, and inferior (arbitral part)/medial (arbitral part)/superior/middle frontal gyrus, and the left postcentral gyrus. Figure 3 shows the details of these regions.

### Relationship between brain network measures and clinical variables

**Figure 4:**
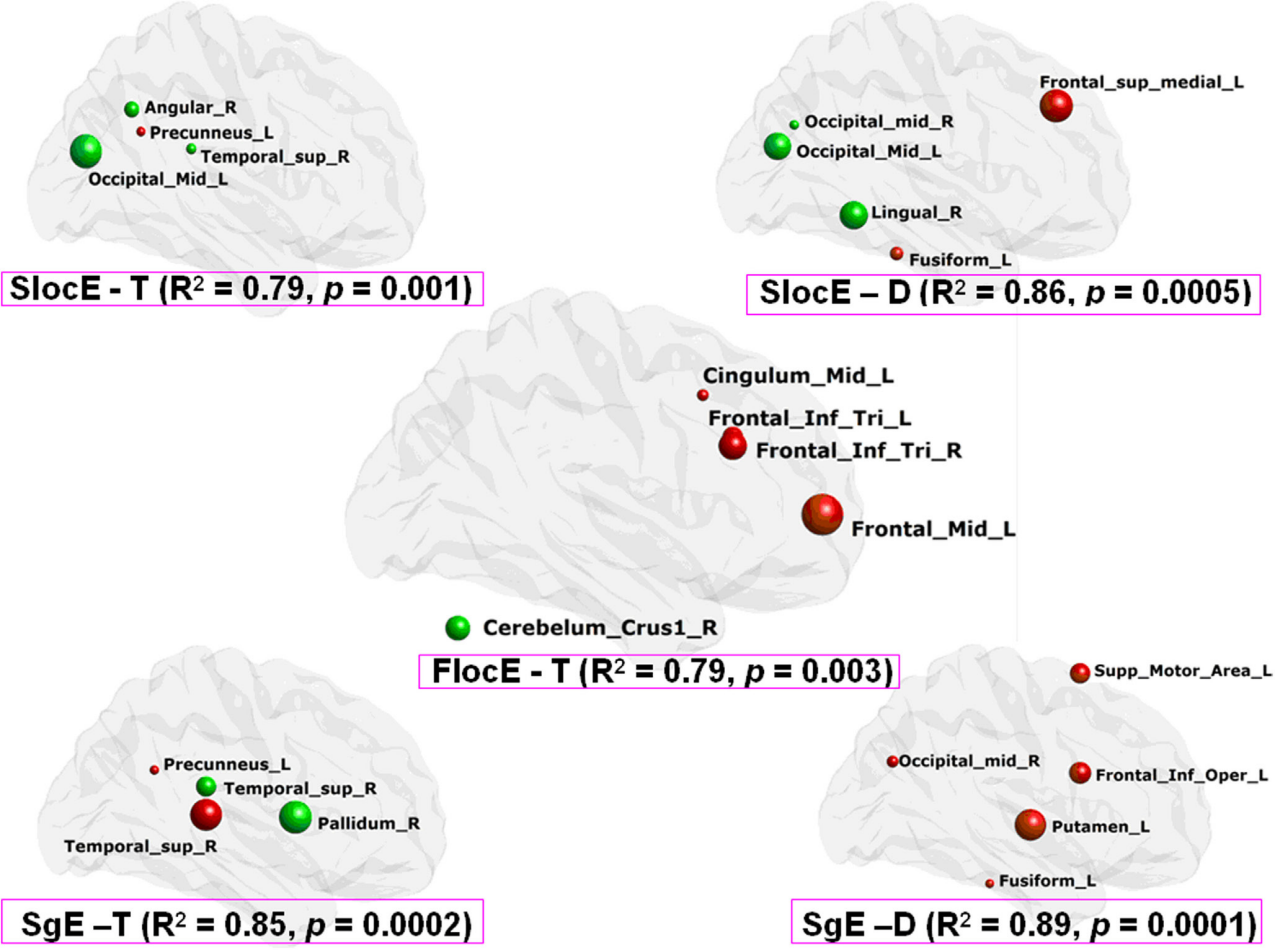
**Clinical performance description from the network nodal efficiency based on MLRM in PD patients**. SlocE, nodal local efficiency of structure network; SgE, nodal global efficiency of structure network; FlocE, nodal local efficiency of functional network; T, tremor degree; D, disease duration. The color of the node reflects the direction of the regression coefficient (i.e., red is positive and green is negative), and the radius of the node reflects the coefficient size.

The normalized network local efficiency of the functional brain network was negatively correlated with the tremor level (*r* = −0.57, *p* = 0.03). We found that the functional brain network nodal local efficiency could effectively describe the variability in tremor performance in the PD patients (*p* = 0.003) (Figure 4). However, we did not find any other salient correlations between the network/nodal properties and clinical performance (i.e., UPDRS and Duration) (all *p* > 0.05).

Moreover, there were no significant correlations between morphological network properties (i.e., local efficiency, global efficiency, gamma, and lambda) and clinical scores (i.e., Tremor, UPDRS, and Duration) (all *p* > 0.05). Despite this result, the morphological network nodal efficiency (i.e., local efficiency and global efficiency) exhibits a high association with the variability of tremor and the duration of PD in the patients (all *p* < 0.001) (Figure 4).

### Association between functional and morphological networks in PD patients

The present study did not find any significant correlation between the functional connectivity of the functional network and the inter-region GM similarity of the morphological network in PD patients (*p* > 0.05). In addition, there were no significant correlations of network efficiency (i.e., local efficiency and global efficiency) between the two types of brain networks in PD patients (all *p* > 0.05).

**Figure 5:**
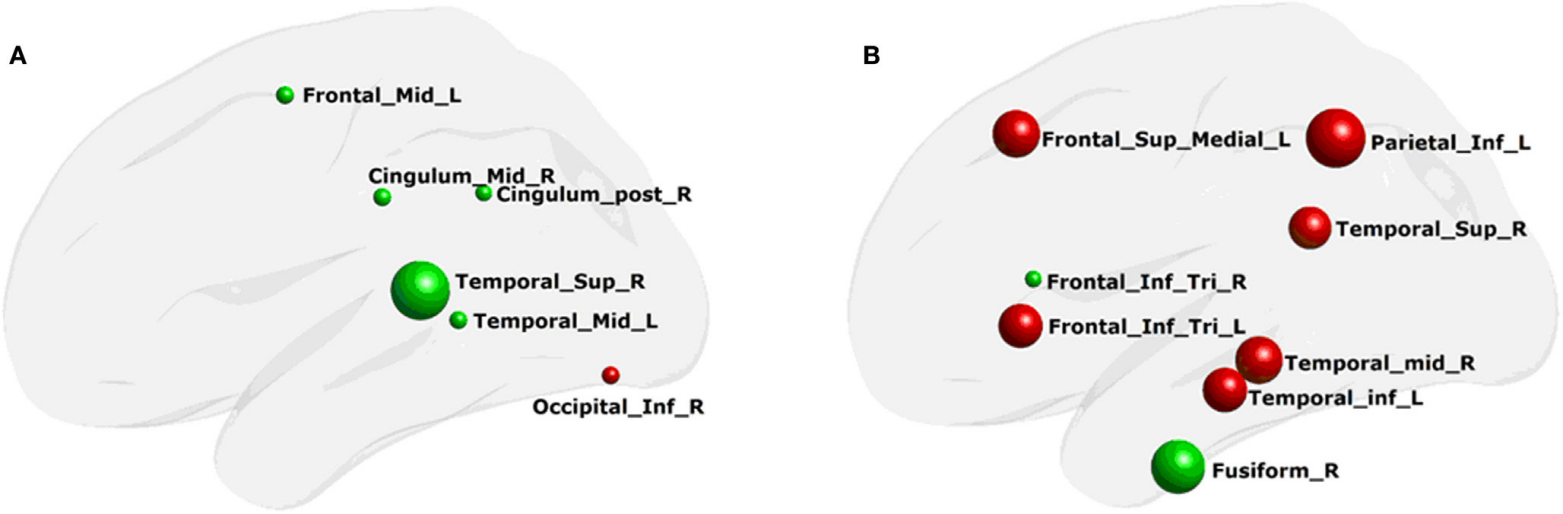
**Regions of network local efficiency in description of the whole-brain network properties**. **(A)** structure network nodal efficiency that could describe the functional network efficiency; **(B)** functional network nodal efficiency that could describe the structure network efficiency. The color of the node and the radius of the node are the same as in Figure 4.

Using MLRM, we found that there was a significant association between the nodal local efficiency in the morphological network and the functional network local efficiency in PD patients. The nodal local efficiency of the regions, including the right superior temporal gyrus, post cingulated cortex, and the inferior occipital gyrus, and the left middle temporal gyrus and middle frontal gyrus (Figure 5A) could describe the functional network local efficiency (*R*^2^ = 0.92, *p* < 0.0002). The functional nodal local efficiency of the regions including the right middle temporal gyrus, superior temporal gyrus, and fusiform, and the left inferior temporal gyrus, inferior parietal gyrus, superior medial frontal gyrus, and the bilateral inferior frontal gyrus (tribal part) (Figure 5B) could also describe the structure network local efficiency (*R*^2^ = 0.84, *p* < 0.03). Notably, these correlations were not found in any aspect of the network global efficiency.

## Discussion

This study explored the topological properties of wavelet-based functional brain networks and individual brain morphological networks in PD patients with tremor and NCs. The main findings can be summarized as follows: (i) decreased network local efficiency and the increased global efficiency was identified in the functional brain network constructed with wavelet scale 2 (i.e., 0.063–0.125 Hz) in PD patients. The network local efficiency instead of the global efficiency performed well in discriminating PD from NCs. Further, there was high correlation between network local efficiency and tremor performance; (ii) the morphological brain network local efficiency and global efficiency were significantly improved in PD patients with tremor. The nodal efficiency of both of these networks performed well in discriminating PD from NCs. Both networks had high correlations with tremor/duration performances. (iii) We identified tight associations of network local efficiency between the functional and morphological networks in PD patients with tremor. These findings provide new insights into the neural substrates related to the pathological damage and the relevant compensation activity in PD patients with tremor.

### Altered functional brain network organization

The wavelet-based functional network analysis method has already been applied to explore the economic properties of the functional brain network in PD patients. The findings of this previous study suggested that the functional brain network in PD patients exhibits a salient decrease in nodal and global efficiency when compared with NCs and that the most significantly changed network organization was observed at wavelet scale 2 in the PD patients (Skidmore et al., 2011). We have noted that there was much evidence to demonstrate the rationality of the above observations in PD patients. Neurotransmitter depletion in the basal ganglia is an important neurochemical characterization in PD patients (Hacker et al., 2012). In fact, dopamine blockade has been shown to affect network efficiency in healthy participants (Achard and Bullmore, 2007), and decreased interregional connectivity (Luo et al., 2014) and network efficiency have been observed in PD patients (Woerner et al., 2009). Thus, the reduced network efficiency in PD patients may reflect the effects of dopamine depletion.

Consistent with this assumption, this study also found that the functional brain network local efficiency within wavelet scale 2 was significantly reduced in PD patients with tremor compared with NCs. Therefore, the decreased network efficiency may be tied to physiological damage in PD patients with tremor. By contrast, we also found that the functional brain network global efficiency was significantly increased in PD patients with tremor compared with NCs. These observations were opposite to the genuine consequence of pathological damage in PD patients. In fact, many previous studies have shown regional hyper-metabolism (Kassubek et al., 2001) and increases in functional connectivity (Helmich et al., 2012) in PD patients with tremor. Despite the essence of these increased connections in PD patients with tremor are still unknown, these increases may be highly associated with cerebral compensation for pathophysiological changes in PD (Zhang et al., 2015).

The results of the multivariate discriminant analysis indicated that the network regional local efficiency pattern could discriminate PD patients from NCs. However, the network regional global efficiency measure could not (Figure 3). These observations suggest that local efficiency rather than global efficiency of functional brain networks contains discriminative information. In addition, we found that the normalized local efficiency (i.e., Gamma) was negatively correlated with the tremor level, which was not observed for network global efficiency. More importantly, the MLRM showed that there was a functional brain network regional local efficiency pattern rather than a global efficiency pattern that could describe the tremor performance in PD patients (Figure 4). The related regions were predominantly located in the cerebellum and frontal cortex. As we know, the cerebellum has been shown to be a critical region involved in the pathophysiology of PD and may play roles in the both the pathological and compensatory effects (Wu and Hallett, 2013). Although the genuine pathological damage of PD occurs at well-recognized subcortical/cerebellum regions [e.g., basal ganglia and the cerebello-thalamo-cortical circuit (Helmich et al., 2012)], recent evidence has highlighted the important roles of cortical regions in understanding PD tremor (Zhang et al., 2015). Of the cerebral regions, the prefrontal cortex has attracted great attention (Taylor et al., 1986; Tsuchiya et al., 2000). Increased neural activity has been found in the dorsolateral prefrontal cortex and prefrontal cortex dysfunction may be a consequence of caudate nucleus dysfunction (Taylor et al., 1986). Consistent with this finding, our previous study has already reported that functional network regional local efficiency performed better than regional global efficiency in discriminating PD subtypes (i.e., PD patients with tremor and those without tremor) from NCs (Zhang et al., 2014). Nevertheless, the observations reported here provide new evidence to suggest that the functional brain network regional local efficiency carries information on the degree of pathology of PD patients.

### Changed morphological brain network organization

Widespread changes in GM volume in PD patients with tremor have been reported in many previous studies (Kassubek et al., 2002; Zhang et al., 2015). To expand these previous findings, this study explored the topological organization of the brain morphological networks in PD patients with tremor. We found that the morphological network local and global efficiency was significantly improved in PD patients with tremor. The improved network efficiency (i.e., local and global efficiency) performed well in discriminating PD from NCs. The improved morphological network efficiency also supported the compensatory interpretation in PD patients with tremor.

More importantly, we also found that the improved morphological network efficiency (i.e., global and local efficiency) could also describe the tremor and duration performances using MLRM. Several clinical measures (i.e., UPDRS, Tremor, and Duration) of PD patients with tremor were measured in this study. However, only tremor behavior and disease duration were observed to be represented in the morphological network local and global efficiency. Thus, in PD patients with tremor, the tremor behavior was tied to the improved morphological network efficiency, which may support a compensatory interpretation. Of course, the compensatory notion in PD with tremor is still little documented. Thus, further studies should focus on the compensation activity in PD patients with tremor. Using morphological network properties may be an important direction to explore the neural substrates underlying PD with tremor.

### Associations of the functional and morphological networks

Considering that the tremor behavior is correlated with the functional and morphological network properties, we further explored the correlation between the functional and morphological networks. We found that morphological network regional local efficiency could describe the functional network local efficiency; and the primary regions were located in the right superior temporal cortex. As mentioned above, the right superior temporal cortex of the morphological network efficiency was correlated with tremor behavior; here, we further found that the morphological network local efficiency in the right superior temporal cortex was highly correlated to functional network local efficiency. Based on these findings, we speculate that tight correlations exist between network local efficiency in the functional and morphological brain networks in PD patients. The tremor performances were represented differently in the functional and morphological brain networks. However, to the best of our knowledge, little evidence has documented the relationship between tremor and brain network properties related to different modalities. This relationship should be deeply investigated in the future researches.

### Limitations

There are several limitations that should be considered in future works. First, this study is still a preliminary exploratory research to detect both structural and functional brain network alterations for PD patients with resting tremor. Although all the patients of this study were PD with resting tremor, the severity levels of the resting tremor varied across the patients and some of them were tremor-dominant while others were not. Recent studies (Prodoehl et al., 2013; Selikhova et al., 2013; Fereshtehnejad et al., 2015) have demonstrated that there may be different neural bases between tremor-dominant PD and PD with tremor or even between PD patients with different levels of tremor. This indicates the necessary of more homogeneous samples in clinical features and phenotypes for understanding the neural basis underlying PD with tremor. However, the small sample size of the present study limited the further analysis on this issue. Therefore, future studies should further investigate the neural basis of the PD patients with different types of tremors (i.e., resting tremor and action/poster tremor), and different levels of tremor in the same types. Second, the asymmetry of tremor may influence the final results. The tremor side- and/or location-specific alterations in PD are often different across patients. The small sample size of the present study limits further analysis of the effects of these confounding factors on our findings. It is necessary in further studies to clarify these important issues. Third, it should be noted that the direct relationship between brain network properties and neurotransmitter reductions is unclear in PD patients with tremor, which may be an important direction of future studies. Fourth, only PD patients at stage I to III in terms of the H–Y were included in the current study to reduce the influence of their motor depicts on the quality of magnetic resonance imaging (MRI) as far as possible. Such a narrow range of the H–Y scores may be not appropriate for the correlation analysis with the network measures. Thereby, the representation of H–Y on the brain network properties in PD patients with tremor should be further explored in future works. Fifth, the findings of the present study were observed during the patients on off medicine condition, the modulation effect of drugs need further explored in other studies.

## Conclusion

In sum, this study provided evidence that the topological organization of the functional brain network (related to the wavelet scale 2) and the individual morphological network are disrupted in PD patients with tremor. There were significant correlations between the functional and morphological networks, which may be correlated with tremor in PD with tremor. These findings provide new insights into the neural basis of PD patients with tremor.

## Conflict of Interest Statement

The authors declare that the research was conducted in the absence of any commercial or financial relationships that could be construed as a potential conflict of interest.

## Footnotes

^1^http://www.fil.ion.ucl.ac.uk/spm/software/spm8/

^2^http://dbm.neuro.uni-jena.de/software/
